# Persistence of Symptoms in Veterans of the First Gulf War: 5-Year Follow-up

**DOI:** 10.1289/ehp.9251

**Published:** 2006-07-06

**Authors:** Gozde Ozakinci, William K. Hallman, Howard M. Kipen

**Affiliations:** 1 Bute Medical School, University of St. Andrews, St. Andrews, Fife, Scotland, United Kingdom; 2 Food Policy Institute, Cook College, Rutgers University, New Brunswick, New Jersey, USA; 3 UMDNJ-Robert Wood Johnson Medical School, EOHSI–Clinical Research and Occupational Medicine Division, Piscataway, New Jersey, USA

**Keywords:** Gulf War illness, medically unexplained symptoms

## Abstract

**Background:**

During the 1990–1991 Gulf War, approximately 700,000 U.S. troops were deployed to the Persian Gulf theater of operations. Of that number, approximately 100,000 have presented medical complaints through various registry and examination programs.

**Objectives:**

Widespread symptomatic illness without defining physical features has been reported among veterans of the 1991 Gulf War. We ascertained changes in symptom status between an initial 1995 symptom evaluation and a follow-up in 2000.

**Methods:**

We assessed mailed symptom survey questionnaires for 390 previously surveyed members of the U.S. Department of Veterans Affairs Gulf War Registry for changes over the 5-year interval in terms of number and severity of symptoms.

**Results:**

For the cohort as a whole, we found no significant changes in symptom number or severity. Those initially more symptomatic in 1995 showed some improvement over time, but remained much more highly symptomatic than those who had lesser initial symptomatology.

**Conclusions:**

The symptom outbreak following the 1991 Gulf War has not abated over time in registry veterans, suggesting substantial need for better understanding and care for these veterans.

During the 1990–1991 Gulf War (GW), approximately 700,000 U.S. troops were deployed to the Persian Gulf theater of operations. Large-scale involvement of U.S. forces in combat in this region largely ended by the winter of 1991. Nevertheless, significant numbers of the 700,000 U.S. service personnel deployed to the region in 1990–1991 presented with medical complaints in the years following operations. Of that number, approximately 100,000 have presented medical complaints through various registry and examination programs (Brown et al. 2002; Stuart et al. 2002). The symptomatology that characterizes their complaints has been described in a number of studies, including investigations from the United Kingdom and other countries (Iowa Persian Gulf Study Group 1997; Lashof and Cassells 1998; Perisan Gulf Veterans Coordinating Board 1995; Unwin et al. 1999). These studies of both randomly sampled and selected populations show that GW veterans clearly report both increased numbers and severity of virtually all symptoms queried when compared with personnel not deployed to the region. Significantly, to date, these symptoms are not reliably associated with characteristic signs or diagnoses of pathological conditions (Eisen et al. 2005; Fukuda et al. 1998; Hodgson and Kipen 1999; Wessely 2004).

Only one study has made an attempt to longitudinally follow GW veterans to determine how their symptomatic presentations have evolved—whether there has been improvement, stability, or decline in the self-reported health of individuals and groups of GW veterans (Hotopf et al. 2003, 2004). This information is critical in trying to understand not just the etiology of such complaints but also how to plan for continued care and rehabilitation.

We have studied, both clinically and by survey, various groups of GW veterans since 1995 (Fiedler et al. 2006; Hallman et al. 2003; Kipen et al. 1999; Peckerman et al. 1999; Pollet et al. 1998). Our original investigations were from a random sample of the Department of Veterans Affairs (VA) GW registry members. We found that physician diagnoses, including those without a generally accepted etiology such as chronic fatigue syndrome, multiple chemical sensitivities, and fibromyalgia, could not account for much of the symptomatology presented and that, based on symptom endorsement and severity, veterans on the registry could be robustly classified as either highly symptomatic (40% of subjects) or mildly symptomatic (60% of subjects) (Hallman et al. 2003). The large number of highly symptomatic “cases” (i.e., self-reported sick individuals), as well as representation from all four service branches, including National Guard and Reserve units, makes the VA registry a robust population from which to investigate the characteristics, although not the prevalence, of GW illnesses. We are currently studying more representative random populations of GW veterans (Fiedler et al. 2006). As part of this random sample of deployed and nondeployed veterans surveyed in 2000, we took advantage of the opportunity to do a 5-year follow-up of our original cohort of individuals from the registry. We hypothesized that the symptom reporting would remain relatively constant over the 5-year frame and that the highly symptomatic cluster would show a greater tendency toward decline in their health status (more symptomatology) after controlling for demographic variables.

## Materials and Methods

### Participants

Participants were U.S. military veterans from the Gulf War Health Registry maintained by the VA. In 1995 (time 1), veterans residing in seven states—Delaware, Illinois, New Jersey, New York, North Carolina, Ohio, and Pennsylvania—were selected by VA’s Environmental Epidemiology Service using a simple random sampling procedure. The veterans were originally selected as part of recruitment for clinical studies of GW illness (Hallman et al. 2003; Nelson et al. 2001; Pollet et al. 1998). A random subset of veterans who completed that survey formed the sampling frame for the present study conducted in 2000 (time 2).

### Procedure

At time 1, we mailed introductory letters and questionnaires with postage-paid response envelopes to 2,011 veterans. We followed this with reminder postcards, a second (identical) letter and questionnaire, and a maximum of three follow-up phone calls at intervals of approximately 2 weeks until a response was received, ultimately yielding 1,161 responses (60%).

At time 2, The U.S. Department of Defense’s Defense Manpower Data Center (DMDC) provided updated addresses and demographic information on those who had responded at time 1. These veterans were sent a letter describing the project and requesting their consent to be interviewed. Records from the Internal Revenue Service and national directory assistance databases (e.g., TeleMatch, Springfield, VA) were used to locate veterans whose letters were returned without forwarding information. These databases did not include cellular telephone numbers.

This 5-year follow-up of the registry cohort was included as a part of a larger investigation of a national random sample of deployed GW veterans (Fiedler et al. 2006). As part of this study, after informed consent was obtained, all subjects were interviewed by a computer-assisted telephone interview (CATI) method. However, to allow direct follow-up comparisons to the data collected by mail at time 1 and to compare data collection modalities, 60% of those in our original registry cohort were randomly mailed a one-page questionnaire, comprising only symptom questions, 2 weeks before their telephone interview (Brewer et al. 2004). The veterans reported an average of five more symptoms via mail than via telephone, mainly as the result of mild symptoms reported by mail that were not reported at all during the telephone interview (Brewer et al. 2004). As a result, we used this one-page written symptom questionnaire as the basis of our comparisons. Additional demographic and other health information was collected as part of the subsequent CATI interview.

### Measures

Participants were asked to examine a list of 48 symptoms identical to that used at time 1 (Table 1). For each symptom, the respondents indicated whether they had experienced “persistent or recurring” problems within the last 6 months, and if so, whether the problems they had experienced were “mild,” “moderate,” or “severe.” For each symptom, a severity index was calculated by coding “no” as 0, “mild” as 1, “moderate” as 2, and “severe” as 3.

Based on factor and cluster analyses of their time 1 symptom data, each respondent had been classified as belonging to one of two clusters: *a*) veterans reporting good health and few moderate/severe symptoms (*n* = 242; ~ 60%), and *b*) veterans reporting fair/poor health and endorsing an average of 37 symptoms, 75% as moderate/severe (*n* = 148; ~ 40%). Thus, there was a total of 390 respondents who completed both the time 1 and time 2 symptomatology assessments (Table 2) (Hallman et al. 2003).

### Statistical analyses

To assess changes in symptoms over time, we conducted paired samples *t-*tests on the average number of symptoms and average severity of symptoms endorsed at time 1 and time 2. We performed repeated measures MANCOVA (multiple analysis of covariance) to examine the main effect of time on the severity of 48 symptoms by controlling for age (mean centered), rank, race, marital status, sex, education, branch of service, duty (active or National Guard/Reserve), smoker status, and cluster membership (“mildly” or “highly” symptomatic as classified at time 1). In addition, we performed an ANCOVA (analysis of covariance) to examine the main effect of cluster membership at time 1 on change in number of symptoms over the 5-year period. The change score was calculated by subtracting time 1 scores from time 2 scores. We used the following variables as controls: age (mean centered), sex, rank, race, marital status, education, branch, duty, and smoking status. For tests of main effects and interactions, we adopted *p* < 0.05 as the critical value. To adjust for multiple comparisons when comparing changes in the 48 individual symptoms, we employed a Bonferroni correction, adopting as a critical value *p* < 0.001 (0.05/48).

## Results

### Response rates

#### Time 1

The results for time 1 have been fully described by Hallman et al. (2003). Of the 1,935 deliverable questionnaires, 1,161 were completed and returned by the respondents, yielding a response rate of 60%. Using chi-square analyses, we tested for potential selection biases and found no significant differences in the distribution of branch of service, duty status (active, Reserve, National Guard), or sex between those randomly selected to be in the sample and those in the registry as a whole.

Logistic regression analyses suggested no significant differences in response rates attributable to sex, date of entry into the registry, branch of service, type of duty (active, Reserve, or National Guard), or rank [enlisted, noncommissioned officer (NCO), warrant officer, officer]. Multivariate analysis of variance also revealed no significant differences in response rates attributable to either specific symptoms or specific diagnoses as determined by VA examining physicians and independent questionnaires completed at the time of registry enrollment (generally, months to years prior to time 1 study).

#### Time 2

Updated DMDC data and data collected by telephone indicated that 70 of the original 1,161 registry veterans were ineligible to participate in the follow-up study. These included veterans who were deceased (*n* = 20), not deployed to the Gulf (*n* = 39), incarcerated (*n* = 3), or incapacitated (e.g., loss of hearing; *n* = 8). Of the remaining 1,091 eligible to participate, 640 (60%) were mailed a questionnaire, of which, 453 (71%) were completed and returned. Forty-five (7%) could not be delivered, and no forwarding address could be found. Of those who completed the questionnaire, 426 were contacted by telephone and by mail, and 398 of these completed both time 1 and time 2 assessments. Thus, 62% of those mailed a questionnaire at time 2 completed it and the subsequent telephone interview.

Eight cases were excluded because of missing data or because the demographic categories to which they belonged were too small to permit meaningful analyses. These included three veterans who served in the Coast Guard, one widowed veteran, three who indicated the “other” category for race, and one for whom rank was unknown, leaving 390 subjects for further analysis.

These 390 respondents did not differ significantly from the 1,161 respondents who participated at time 1 in terms of sex (8.5% females vs. 9.6% of total); rank (12.6% officers vs. 10.1% of total); duty status at mobilization (57.5% active vs. 59.0% of total); or branch (70.0% Army vs. 70.2% of total). They were significantly different from the 1,161 originally studied in terms of age at time 2 (42.7 years vs. 41.7 years for total), racial composition (16.9% African American vs. 23.2% of total; *p* < 0.006, two-sided), and education (22.8% college graduates vs. 19.1% of total; *p* < 0.02). In addition, *t*-tests with Bonferroni correction revealed no significant differences in the number of symptoms or their average severity reported at time 1 by the 390 current respondents and the remainder of the time 1 cohort.

### Change in number of reported symptoms over time

At time 1, the mean number of symptoms (± SD) reported by the sample of 390 was 22.07 ± 12.92. At time 2, the mean number of symptoms increased slightly (0.67 symptoms) to 22.74 ± 12.89; however, this change was not significant [*t*(389) = −1.48; *p* = 0.14]. Average symptom severity also increased slightly from 0.83 ± 0.62 at time 1 to 0.84 ± 0.62 at time 2. Again, this change was not significant [*t*(398) = −0.52; *p* = 0.61]. Results of ANCOVA on change in number of symptoms controlling for age (mean centered), sex, rank, race, marital status, education, branch, duty, and smoking status revealed that the effect of time 1 cluster membership was significant [*F*(1, 379) = 31.84; *p* < 0.001] (Table 3). Those veterans classified as mildly symptomatic at time 1 showed an increase in number of symptoms over time (mean, 2.33), and those veterans who were highly symptomatic showed a decrease in symptoms at time 2 (mean, −2.04). Also, there were significant effects of race [*F*(1, 379) = 15.11; *p* < 0.001] and age [*F*(1, 379) = 6.98; *p* < 0.05]. Black veterans reported 4.26 more symptoms at time 2, whereas white veterans showed a slight decrease [*t* (388) = −3.62; *p* < 0.001]. Increase in the number of symptoms over time was associated with older age (*r* = 0.12; *p* < 0 .05).

### Changes in symptom severity over time

A repeated measures MANCOVA indicated that there was no significant difference over time in the severity of the 48 symptoms. However, we observed multivariate effects on severity for age [Wilks’s Λ= 0.756; *F*(48, 332) = 2.24; *p* < 0.001], race [Wilks’s Λ= 0.803; *F* (48, 332) = 1.70; *p* < 0.005], and cluster membership [Wilks’s Λ= 0.344; *F*(48, 332) = 13.19; *p* < 0.001] (Table 1). In addition, there was a significant time × smoker status effect [Wilks’s Λ = 0.815; *F*(48, 332) = 1.57; *p* < 0.05] and a significant time × cluster membership effect [Wilks’s Λ= 0.790; *F* (48, 332) = 1.84; *p* < 0.005].

Next, we looked at tests of between-subject effects for the variables that yielded significant multivariate tests to examine the severity changes in individual symptoms. The main effect of age was significant for “persistent or recurring problems” with “arms, hands, and shoulders,” “back problems,” “frequent or painful urination,” “sexual or genital problems,” “pain in arms or legs,” and “pain in more than one joint,” with the severity of all these symptoms increasing more in older individuals. The main effect of race was significant for “persistent or recurring problems” with “constipation,” “hair,” and “sweating not due to exercise,” with all three symptoms increasing more in severity among African-American veterans (Table 4). The main effect of cluster membership was significant for “persistent or recurring problems” with all 48 symptoms at the *p* < 0.001 level, with *F*(1, 379) values ranging from 32.96 to 329.59. Veterans who were highly symptomatic at time 1 had higher severity scores at time 2 than those who reported mild symptomatology.

The time × smoker status interaction term revealed that it was significant for “persistent or recurring problems” with “mouth, teeth, gums” (Table 4). Paired samples *t*-tests showed that while the changes in this symptom were not significant for those who never smoked and for former smokers, the increase in symptom severity for the current smokers was significant [*t*(100) = −3.37; *p* < 0.005]. The time × cluster membership interaction term was significant for “persistent or recurring problems,” with 18 symptoms listed in Table 4. Paired samples *t*-tests for 18 symptoms for the cluster originally reporting mild symptomatology showed that after Bonferroni correction (0.05/18 = 0.002), the increases over time in the severity of “abdominal gas,” “muscle aches or cramps,” “numbness or tingling sensations,” “pain in arms or legs,” and “difficulty concentrating” were significant. For the cluster that reported high symptomatology at time 1, the decreases in the severity of “headaches,” “difficulty breathing,” “unrefreshing sleep,” “sleeping more than usual,” “sudden mood changes,” and “feeling depressed or blue” were significant after Bonferroni correction (means ± SDs presented in Table 1).

## Discussion

Consistent with our hypothesis, this group of Gulf War Registry veterans continued to experience significant symptoms 10 years after deployment to the Middle East, > 5 years after they presented for registry examination. In the cohort as a whole, we found no significant changes in individually matched symptom reporting, neither improvement nor progression, in number or severity of symptoms. However, some subgroups did show significant, albeit relatively small, changes. Important predictors of worsening included being less symptomatic at time 1, African-American race, older age, and being a smoker at time 2. The effects of older age and smoking seem logical based on their expected impact on musculoskeletal changes and on oral health, respectively. However the effects of race and cluster are less straightforward.

Those who were classified as highly symptomatic at time 1 had higher severity scores compared with those who were mildly symptomatic at both times. However, for 18 symptoms we did observe a time × cluster effect; this time × cluster effect showed that a subset of symptoms in mildly symptomatic people increased severity over time, whereas a distinct subset of symptoms in highly symptomatic people decreased severity over time. In spite of the increase in symptom number and severity for the mild cluster and the decrease for the high cluster, the individuals in the two groups still remain substantially apart and distinct, with high individuals maintaining high symptomatology over time.

A history of physician diagnoses did not predict symptoms at time 1 (Hallman et al. 2003), so we did not include this data in these subsequent analyses. Prior medical evaluations of GW veterans have not demonstrated explanatory medical findings or diagnoses between those with and without symptoms (Eisen et al. 2005; Fukuda et al. 1998; Hodgson and Kipen 1999; Wessely 2004).

Controlled trials of behavioral therapy and antibiotics for Gulf War illness have been conducted, and some of our subjects likely participated in both routine and experimental therapies. However, based on reports from therapeutic trials to date, there is little reason to expect a large effect of treatment on symptoms (Donta 2004; Wessely 2004).

Hotopf et al. (2003) measured total symptoms in a random-sample U.K. Gulf War veteran cohort in 1997 and again in 2001. They reported a nonsignificant decrease in symptoms over the 4 years, consistent with our finding of only minor variation. Using a fatigue scale, a psychiatric distress scale (General Health Questionnaire) and a measure of physical function (Medical Outcome Study Short-Form 36), Hotopf et al. (2004) reported that greater initial severity of fatigue predicted greater severity for all three outcomes over a 5-year follow-up. This is somewhat contrary to our findings that more symptomatic individuals actually showed greater declines in symptomatology. This difference may be explained by their use of scales for specific conditions rather than unaggregated symptom data, as we used. They also found that the attribution factor “Belief in Gulf War Syndrome” was associated with worsening, which is consistent with our previous findings at time 1 (Boyd et al. 2003).

### Limitations

The veterans we studied were taken from a registry and thus may not be completely representative of all deployed soldiers. We did not have a comparison group that was not deployed in this analysis, but we used individual matching to avoid many sources of bias, and all analyses were adjusted for multiple potential demographic confounders. Self-report items are always subject to bias; also, the interpretation of symptoms in the absence of explanatory diagnoses remains a challenge, but cannot be avoided until more reliable biomarkers of such illnesses are developed.

### Implications

Overall, we found little change to indicate either a self-limited condition or one characterized by significant progression. Although this does not have clear implications in terms of etiology, it does suggest that continued efforts to find effective treatments and understand etiologies(s) are relevant in the absence of substantial improvement in the severity and number of symptoms experienced by Gulf War veterans over time.

## Figures and Tables

**Table 1:** Severity (mean ± SD) of 48 symptoms at time 1 and time 2 for highly symptomatic and mildly symptomatic clusters and overall means.

	Mild		High		
Symptom	Time 1 (*n* = 242)	Time 2 (*n* = 242)	Time 1 (*n* = 148)	Time 2 (*n* = 148)	Overall mean Time 1/time 2
Headaches	0.85 ± 0.98	0.85 ± 0.97	2.11 ± 0.91	1.77 ± 0.98*	1.33/1.20
Eyes or vision	0.51 ± 0.79	0.67 ± 0.83	1.36 ± 0.94	1.45 ± 0.94	0.83/0.96
Ears or hearing	0.55 ± 0.83	0.69 ± 0.89	1.37 ± 1.05	1.30 ± 1.01	0.86/0.93
Nose or sinuses	0.86 ± 1.02	0.94 ± 1.01	1.86 ± 1.07	1.74 ± 1.11	1.24/1.25
Multiple chemical sensitivities	0.39 ± 0.74	0.51 ± 0.86	1.51 ± 1.12	1.57 ± 1.17	0.81/0.92
Mouth, teeth, and gums	0.41 ± 0.75	0.50 ± 0.83	1.25 ± 1.11	1.22 ± 1.08	0.73/0.77
Ability to taste	0.06 ± 0.26	0.12 ± 0.39	0.69 ± 0.90	0.70 ± 0.89	0.30/0.34
Difficulty swallowing	0.11 ± 0.41	0.21 ± 0.56	0.72 ± 0.85	0.73 ± 0.93	0.34/0.41
Throat problems	0.22 ± 0.55	0.26 ± 0.64	1.03 ± 1.04	0.84 ± 0.94	0.53/0.48
Swollen glands	0.18 ± 0.46	0.25 ± 0.58	1.01 ± 1.05	0.79 ± 0.94	0.49/0.46
Difficulty breathing	0.33 ± 0.66	0.36 ± 0.69	1.45 ± 1.00	1.03 ± 0.95*	0.75/0.62
Coughing	0.38 ± 0.65	0.46 ± 0.74	1.39 ± 1.07	1.21 ± 1.06	0.76/0.74
Chest discomfort	0.43 ± 0.74	0.48 ± 0.75	1.56 ± 0.94	1.33 ± 1.01	0.86/0.80
Irregular heartbeat	0.16 ± 0.52	0.29 ± 0.62	0.71 ± 1.03	0.71 ± 0.91	0.37/0.45
Arms, hands, and shoulders	0.55 ± 0.87	0.67 ± 0.87	1.82 ± 1.08	1.63 ± 1.03	1.03/1.03
Back problems	0.70 ± 0.90	0.86 ± 0.98	1.78 ± 1.01	1.75 ± 1.11	1.11/1.20
Nausea	0.18 ± 0.45	0.21 ± 0.56	0.99 ± 0.95	0.84 ± 0.93	0.49/0.45
Vomiting	0.05 ± 0.25	0.09 ± 0.40	0.47 ± 0.77	0.39 ± 0.73	0.21/0.20
Stomach or digestive problems	0.52 ± 0.86	0.69 ± 0.93	1.59 ± 1.07	1.53 ± 1.08	0.93/1.01
Diarrhea	0.39 ± 0.73	0.48 ± 0.80	1.29 ± 1.08	1.21 ± 1.08	0.73/0.76
Constipation	0.17 ± 0.51	0.24 ± 0.57	0.61 ± 0.85	0.74 ± 0.95	0.33/0.43
Abdominal gas	0.54 ± 0.81	0.72 ± 0.95*	1.74 ± 1.05	1.54 ± 1.10	0.99/1.03
Abdominal pain	0.29 ± 0.66	0.42 ± 0.74	1.41 ± 0.98	1.26 ± 1.04	0.72/0.74
Frequent or painful urination	0.16 ± 0.46	0.24 ± 0.66	0.74 ± 0.95	0.69 ± 0.90	0.38/0.41
Muscle aches or cramps	0.57 ± 0.81	0.82 ± 0.93*	1.95 ± 0.86	1.81 ± 1.06	1.09/1.19
Sexual or genital problems	0.21 ± 0.52	0.28 ± 0.67	0.97 ± 1.12	0.93 ± 1.10	0.50/0.53
Numbness or tingling	0.47 ± 0.73	0.70 ± 0.86*	1.80 ± 0.89	1.72 ± 1.08	0.97/1.08
Pain in arms and legs	0.57 ± 0.82	0.79 ± 0.95*	2.11 ± 0.87	1.88 ± 1.06	1.15/1.20
Pain in more than one joint	0.71 ± 0.92	0.86 ± 0.99	2.17 ± 0.88	2.05 ± 1.00	1.27/1.31
Skin (including rashes)	0.74 ± 0.95	0.68 ± 0.98	1.69 ± 1.12	1.37 ± 1.15	1.10/0.94
Hair problems	0.18 ± 0.51	0.21 ± 0.61	0.81 ± 1.03	0.72 ± 1.00	0.42/0.40
Cuts or sores	0.34 ± 0.66	0.33 ± 0.70	1.04 ± 1.06	0.91 ± 1.06	0.61/0.55
Fainting spells	0.05 ± 0.26	0.06 ± 0.29	0.32 ± 0.73	0.24 ± 0.56	0.15/0.13
Losing balance/dizziness	0.38 ± 0.64	0.52 ± 0.77	1.43 ± 1.04	1.27 ± 0.93	0.78/0.80
Difficulty concentrating	0.62 ± 0.81	0.81 ± 0.89*	1.99 ± 0.90	1.87 ± 0.91	1.14/1.21
Difficulty remembering	0.81 ± 0.90	1.04 ± 0.94	2.13 ± 0.88	2.11 ± 0.94	1.31/1.45
Feeling sickly	0.31 ± 0.59	0.42 ± 0.74	1.59 ± 0.98	1.37 ± 0.99	0.80/0.78
Unrefreshing sleep	1.01 ± 0.95	1.14 ± 0.97	2.48 ± 0.73	2.26 ± 0.83*	1.57/1.57
Sleeping more than usual	0.54 ± 0.82	0.54 ± 0.86	1.62 ± 1.21	1.28 ± 1.20*	0.95/0.82
Fatigue (not from exercise)	0.92 ± 0.87	1.04 ± 0.98	2.34 ± 0.81	2.18 ± 0.81	1.46/1.47
Fatigue after exercise	0.37 ± 0.71	0.55 ± 0.86	1.82 ± 1.06	1.74 ± 1.13	0.92/1.00
Unexplained weakness	0.39 ± 0.64	0.48 ± 0.79	1.91 ± 0.98	1.72 ± 1.04	0.96/0.95
Feeling anxious	0.61 ± 0.76	0.71 ± 0.91	2.00 ± 0.95	1.74 ± 1.01	1.14/1.10
Sudden mood changes	0.64 ± 0.81	0.72 ± 0.90	2.11 ± 1.02	1.80 ± 1.07*	1.20/1.13
Sensitivity to heat or cold	0.37 ± 0.71	0.44 ± 0.80	1.49 ± 1.18	1.34 ± 1.18	0.79/0.78
Fever or chills	0.14 ± 0.42	0.19 ± 0.52	1.09 ± 1.05	0.84 ± 0.92	0.50/0.44
Sweating	0.31 ± 0.73	0.48 ± 0.84	1.39 ± 1.10	1.28 ± 1.11	0.72/0.79
Feeling depressed or blue	0.59 ± 0.80	0.71 ± 0.98	1.95 ± 1.00	1.61 ± 1.09*	1.11/1.05

* Significantly different from time 1 to time 2 within highly symptomatic and mildly symptomatic clusters.

**Table 2:** Demographic characteristics of the sample (*n* = 390).

Demographic variable	No. (%)
Sex	
Male	364 (93.3)
Female	26 (6.7)
Military branch	
Army	267 (68.5)
Air Force	35 (9.0)
Marines	64 (16.4)
Navy	24 (6.2)
Education	
High school	108 (27.7)
College	231 (59.2)
Graduate school	51 (13.1)
Duty	
Active duty	227 (58.2)
National Guard	72 (18.5)
Reserves	91 (23.3)
Race	
White	324 (83.1)
Black	66 (16.9)
Marital status	
Married	217 (55.6)
Divorced	19 (4.9)
Separated	13 (3.3)
Single, never married	132 (33.8)
Not married but living with mate	9 (2.3)
Rank	
Enlisted	183 (46.9)
NCO	157 (40.3)
Warrant officer	8 (2.1)
Commissioned officer	42 (10.8)
Smoker	
Never smoked	178 (45.6)
Former smoker	111 (28.5)
Current smoker	101 (25.9)

**Table 3:** Number of symptoms (mean ± SD) at time 1 and time 2 across demographic variables and cluster membership.

Demographic variable	Time 1	Time 2
Cluster membership		
Mildly symptomatic	14.22 ± 8.67	16.55 ± 10.49
Highly symptomatic	34.90 ± 7.15	32.86 ± 9.64
Sex		
Male	22.02 ± 12.96	22.81 ± 12.96
Female	22.73 ± 12.57	21.81 ± 12.03
Military branch		
Army	23.47 ± 12.46	24.14 ± 12.76
Air Force	23.60 ± 12.39	23.66 ± 12.09
Marines	16.72 ± 13.86	18.14 ± 13.44
Navy	18.54 ± 12.44	18.13 ± 10.55
Education		
High school	23.62 ± 13.12	24.31 ± 12.79
College	22.45 ± 12.95	23.42 ± 13.01
Graduate school	17.04 ± 11.24	16.33 ± 10.65
Duty		
Active duty	22.77 ± 13.22	23.26 ± 12.93
National Guard	21.42 ± 11.78	23.04 ± 11.93
Reserves	20.83 ± 13.01	21.20 ± 13.50
Race		
White	21.47 ± 12.68	21.41 ± 12.45
Black	25.00 ± 13.77	29.26 ± 13.12
Marital status		
Married	22.77 ± 13.18	23.75 ± 13.17
Divorced	25.53 ± 13.79	25.26 ± 13.77
Separated	21.00 ± 13.48	23.62 ± 13.74
Single, never married	20.64 ± 12.26	20.40 ± 12.14
Not married but living with mate	20.22 ± 13.34	26.11 ± 11.21
Rank		
Enlisted	21.54 ± 12.99	21.71 ± 12.69
NCO	23.85 ± 12.82	25.50 ± 13.14
Warrant officer	19.13 ± 16.14	22.25 ± 13.81
Full officer	18.29 ± 11.56	17.00 ± 10.16
Smoker		
Never smoked	20.92 ± 13.15	20.87 ± 13.12
Former smoker	21.24 ± 13.17	23.19 ± 12.69
Current smoker	25.00 ± 11.85	25.54 ± 12.25

**Table 4:** Significant main and interaction effects (except the main effect of cluster membership) for change in symptom severity.

Source	*F*(1, 379)	*p*-Value
Age		
Arms, hands, shoulders	21.29	0.000
Back problems	11.64	0.001
Frequent or painful urination	11.88	0.001
Sexual or genital problems	22.07	0.000
Pain in arms or legs	12.28	0.001
Pain in more than one joint	13.60	0.000
Race		
Constipation	13.98	0.000
Hair	11.05	0.001
Sweating not due to exercise	10.70	0.001
Time × smoker status		
Mouth, teeth, gums	11.29	0.001
Time × cluster membership		
Headaches	12.45	0.000
Swollen glands in neck or armpit	10.81	0.001
Difficulty breathing	23.69	0.000
Abdominal gas	15.72	0.000
Abdominal pain	10.68	0.001
Muscle aches or cramps	18.20	0.000
Numbness or tingling sensations	11.52	0.001
Pain in arms or legs	21.78	0.000
Difficulty concentrating	10.85	0.001
Feeling sickly	14.40	0.000
Unrefreshing sleep	13.84	0.000
Sleeping more than usual	12.58	0.000
Unexplained weakness	13.19	0.000
Feeling anxious or upset	16.49	0.000
Sudden mood changes	20.62	0.000
Fever or chills	15.61	0.000
Sweating not due to exercise	12.14	0.001
Feeling depressed or blue	22.87	0.000
